# Efficacy and safety of inorganic nitrate/nitrite supplementary therapy in heart failure with preserved ejection fraction

**DOI:** 10.3389/fcvm.2023.1054666

**Published:** 2023-02-02

**Authors:** Feng Lv, Junyi Zhang, Yuan Tao

**Affiliations:** ^1^Department of Cardiology, Shengzhou People’s Hospital (The First Affiliated Hospital of Zhejiang University Shengzhou Branch), Shengzhou City, Zhejiang Province, China; ^2^Department of Cardiology, Dushu Lake Hospital Affiliated to Soochow University, Suzhou City, Jiangsu Province, China

**Keywords:** heart failure with preserved ejection fraction (HFpEF), inorganic nitrate, inorganic nitrite, meta-analysis, exercise capacity

## Abstract

**Background:**

Approximately half of patients with heart failure have a preserved ejection fraction (HFpEF). To date, only SGLT-2i, ARNi, and MRAs treatments have been shown to be effective for HFpEF. Exercise intolerance is the primary clinical feature of HFpEF. The aim of this meta-analysis was to explore the effect of inorganic nitrate/nitrite supplementary therapy on the exercise capacity of HFpEF patients.

**Methods:**

We searched PubMed, Embase, Cochrane Library, OVID, and Web of Science for eligible studies for this meta-analysis. The primary outcomes were peak oxygen consumption (peak VO_2_), exercise time, and respiratory exchange ratio (RER) during exercise. The secondary outcomes were cardiac output, heart rate, systolic blood pressure, diastolic blood pressure, mean arterial pressure, and systemic vascular resistance during rest and exercise, respectively.

**Results:**

A total of eight randomized-controlled trials were enrolled for this meta-analysis. We found no benefit of inorganic nitrate/nitrite on exercise capacity in patients with HFpEF. Inorganic nitrate/nitrite compared to placebo, did not significantly increased peak VO_2_ (MD = 0.361, 95% CI = −0.17 to 0.89, *p* = 0.183), exercise time (MD = 9.74, 95% CI = −46.47 to 65.95, *p* = 0.734), and respiratory exchange ratio during exercise (MD = −0.003, 95% CI = −0.036 to 0.029, *p* = 0.834). Among the six diameters reflecting cardiac and artery hemodynamics, inorganic nitrate/nitrite can lower rest SBP, rest/exercise DBP, rest/exercise MAP, and exercise SVR, but has no effect in cardiac output and heart rate for HFpEF patients.

**Conclusion:**

Our meta-analysis suggested that inorganic nitrate/nitrite supplementary therapy has no benefit in improving the exercise capacity of patients with HFpEF, but can yield a blood pressure lowering effect, especially during exercise.

## Background

Approximately half of patients with heart failure have a preserved ejection fraction (HFpEF) (1, 2). HFpEF occurs almost exclusively in older population, and many are asymptomatic at rest and show abnormalities only during exercise (3–5). To date, only SGLT-2i, ARNi, and MRAs treatments have been shown to be effective for HFpEF (6–8).

Exercise intolerance is the primary clinical feature of HFpEF and is responsible for the severely reduced quality of life of these patients (9–12). However, the mechanism of this limitation has not been understood completely. Compared with heart failure with reduced ejection fraction, HFpEF has a distinct pathophysiology characterized by ventricular diastolic dysfunction. During exercise, a normally functioning left ventricle can diastole to a larger volume with no increase in filling pressure, but in individuals with HFpEF, left ventricle filling pressure with exercise increases remarkably, producing symptoms of dyspnea (13–16). Not only have diastolic dysfunction been identified, but evidence exists for abnormalities in peripheral arteries and skeletal muscle. The impaired exercise vasodilatory reserve and reduction in skeletal muscle perfusion may contribute significantly to exercise intolerance of HFpEF patients (17–19). Multiple lines of evidence suggest that the impaired perfusion results are due in large part to low availabilities in nitric oxide (NO)-cyclic guanosine monophosphate (cGMP) signaling (18, 20).

Traditionally, endogenous NO was thought to generate exclusively by NO synthases (21). In recent years, however, the nitrate–nitrite–NO pathway has been recognized as an important alternative *in vivo* source of NO (22, 23). Intriguingly, tissue hypoxia and acidosis can enhance the reduction of nitrite to NO, a condition that exercise develops. This suggests that nitrate/nitrite might better target hemodynamic derangements developed during exercise in people with HFpEF (24–27). Moreover, compared to organic nitrates such as isosorbide mononitrate and dinitrate, inorganic nitrate/nitrite is less likely to cause hypotension/headache and rarely develops tolerance (28, 29), which provides a considerable promise for the use of inorganic nitrate/nitrite in the treatment of HFpEF.

Multiple randomized controlled studies have investigated the effect of inorganic nitrate/nitrite on exercise capacity and cardiac hemodynamics in patients with HFpEF, with the administration duration ranging from acute to short-term. Interventions of these studies were also various, including intravenous sodium nitrite, inhaled nitrite, oral potassium nitrate, and NO_3_-rich beetroot juice (BRJ). However, the conclusions were inconsistent. Some studies demonstrated a positive effect of inorganic nitrate/nitrite in exercise capacity in patients with HFpEF (21, 25, 26, 30), while others did not (28, 29, 31, 32). To date, no study has summarized the results of relevant trials and thus the conclusion is unclear. Accordingly, we conducted the current meta-analysis of randomized controlled trials to explore the clinical viability of inorganic nitrate/nitrite on exercise performance and cardiac hemodynamics in patients with HFpEF, providing clinicians with new thoughts of HFpEF pharmacological therapeutics.

## Methods

### Search strategy

A systematic search was conducted on PubMed, Embase, Cochrane Library, OVID, and Web of Science for eligible studies published up to December 31, 2020 using the following search terms: (“nitrate” or “azotate” or “nitrite”) and (“heart failure” or “cardiac failure” or “heart decompensation”) and (“preserved” or “normal”). Similar searches were made on clinicaltrials.gov to ensure no bias caused by unpublished trials. We also manually screened the reference lists of key articles to further identify potential eligible studies. There is no restriction in primary outcomes or language.

### Inclusion and exclusion criteria

The inclusion criteria are as follows: (1) study design: randomized controlled trial, (2) study population: patients diagnosed with HFpEF, (3) intervention: inorganic nitrate/nitrite, (4) comparator: placebo or control, and (5) reported outcomes of exercise capacity or cardiac hemodynamics. HFpEF was defined as symptoms of chronic heart failure (dyspnea and/or fatigue) and preserved left ventricular ejection fraction (≥50%). Three indicators of exercise capacity were considered as the primary outcomes: peak oxygen consumption (peak VO_2_), respiratory exchange ratio (VCO_2_/VO_2_) during exercise and exercise time. The secondary outcomes were parameters of cardiac and arterial hemodynamics, including cardiac output, heart rate, systolic blood pressure, diastolic blood pressure, mean arterial pressure, and systemic vascular resistance at rest and exercise, respectively. Studies that did not reported any of the outcomes mentioned above were excluded. Two investigators (Lv-F, Zhang-JY) independently reviewed the titles and abstracts of studies identified by the search strategy, and studies that satisfied the inclusion criteria were entered into the full-text assessment. When articles were only abstracts, efforts were made to contact the authors for full-text, if failed, we would eventually exclude these articles due to the insufficient data and potential significant bias.

### Quality evaluation and data extraction

All included studies were evaluated for quality by two investigators independently, with disagreements resolved by discussion. The risk of bias was assessed using the criteria proposed by the Cochrane back review group (33). The level of evidence was assessed based on the guidelines of the GRADE working group (34).

The following data were extracted from each selected study: RCT design (parallel or crossover), number of participants per arm, nature of intervention, exercise pattern, age, sex, race, cardiac function, complication, basic medication, and outcomes of interest before and after intervention.

### Statistical analysis

Data were pooled in a meta-analysis in the forms of forest plots. Given that all the outcomes were continuous variables, the combined estimates were presented as mean difference (MD) and 95% CI. If the units are not uniform, a standard mean difference (SMD) will be used. Heterogeneity between studies was assessed using Chi square test and magnitude by calculating *I*^2^ statistic, with *I*^2^ > 50% regarded as indicating moderate-to-high heterogeneity (35). A random-effect or fixed-effect model was used depending on the heterogeneity calculated. A sensitivity analysis was performed by excluding one study each time, in order to evaluate the effect of single study on the overall estimates. Publication bias was assessed by constructing a funnel plot of each study’s effect size against the standard error. The funnel plot asymmetry was assessed using Begg and Egger’s tests, with a *p*-value <0.1 considered as significant publication bias. We also used the trim-and-fill computation to estimate the impact of publication bias on the interpretation of results (36). All statistical tests were performed with Stata (version 12.0).

## Results

### Search results and study characteristics

**Figure 1:**
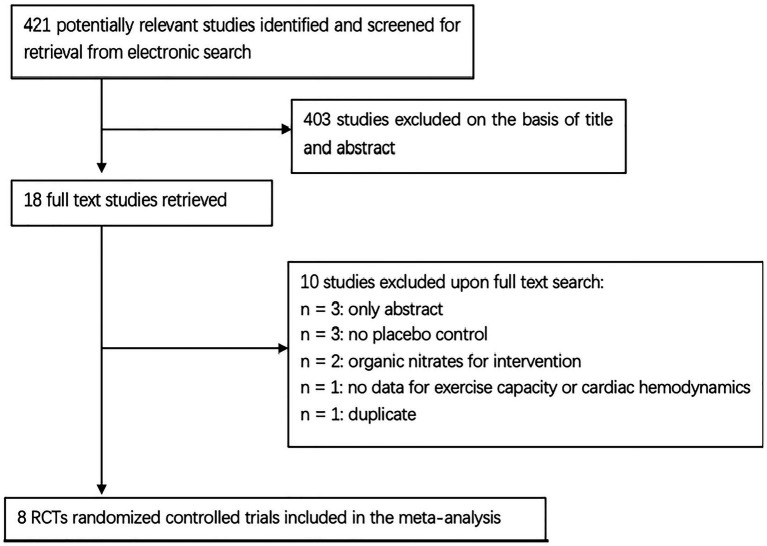
Flow diagram of the study selection process.

Through a literature searching, we identified 421 studies, of which eight RCTs with 335 patients were eventually included in the current meta-analysis (Figure 1). The eight RCTs were all published after 2014, comparing the effect of inorganic nitrate/nitrite on HFpEF with that of placebo (21, 25, 26, 28–32). Four trials were parallel-group design with baseline characteristics well matched in two arms (25, 26, 30, 32). The other four were cross-over design, meaning that the baseline characteristics in two arms were exactly the same (21, 28, 29, 31). Four trials (25, 26, 28, 30) used sodium nitrite (intravenous or inhaled) as intervention and the other four (21, 29, 31, 32) used nitrate-rich beetroot juice (BRJ) as intervention. Five trials (21, 25, 26, 29, 30) looked at the acute effects of nitrate/nitrite while other threes (28, 31, 32) investigated the short-term effects (≥1 week). One trial (32) compared BRJ with placebo on a background of supervised exercise. The exercise pattern for testing varied among studies, including maximal-effort exercise, submaximal-effort exercise (20/45-W workload) and upright cycle ergometry. The detailed characteristics of the eight studies are shown in Table 1.

**Table 1:** Characteristics of studies included in the meta-analysis.

	Borlaug 2015 (25)		Zamani 2015 (21)		Borlaug 2016 (26)		Eggebeen 2016 (31)	
	Treatment (*n* = 14)	Control (*n* = 14)	Treatment (*n* = 17)	Control (*n* = 17)	Treatment (*n* = 13)	Control (*n* = 13)	Treatment (*n* = 20)	Control (*n* = 20)
RCT design	Parallel-group		Cross-over		Parallel-group		Cross-over	
Intervention	A single, acute dose of intravenous sodium nitrite (50 mg/kg/min for 5 min)		A single, acute dose of BRJ (12.9 mmol NO3^−^ in 140 ml)		A single, acute dose of nebulized inhaled sodium nitrite (90 mg)		A daily dose of BRJ (6.1 mmol of NO3^−^ in 70 ml) for 1 week	
Intervention drug	Nitrite		Nitrate		Nitrite		Nitrate	
Control	Placebo		Placebo		Placebo		Placebo	
Treatment duration	Acute		Acute		Acute		Short-term	
Exercise pattern	Constant 20-W exercise		Maximal-effort exercise		Constant 20-W exercise		Constant 45-W exercise	
White race, %	93	100	18	18	NA	NA	60	60
Age, yrs	70 ± 8	69 ± 6	65.5 ± 8.9	65.5 ± 8.9	67 ± 9	72 ± 10	69 ± 6.8	69 ± 6.8
Male, %	36	43	88	88	54	38	15	15
BMI, kg/m^2^	32 ± 7.0	33.4 ± 6.6	35.4 ± 5.4	35.4 ± 5.4	33.2 ± 2.9	30.8 ± 5.2	32.9 ± 5.6	32.9 ± 5.6
NYHA class, %								
II	NA	NA	71	71	NA	NA	70	70
III	NA	NA	24	24	NA	NA	30	30
HTN, %	86	79	100	100	77	85	100	100
DM, %	21	21	71	71	31	31	35	35
CHD, %	29	43	18	18	54	54	NA	NA
Drug therapy, %								
Diuretic	43	50	59	59	38	62	65	65
ACEI/ARB	79	43	65	65	69	54	65	65
β-Blocker	NA	NA	65	65	31	54	25	25
Spironolactone	NA	NA	6	6	NA	NA	NA	NA
CCB	NA	NA	41	41	NA	NA	35	35
Statin	57	50	59	59	23	38	NA	NA
NT-proBNP, pg/ml	249 (118–890)	585 (175–1,575)	144 (60.3–192.0)	144 (60.3–192.0)	551 (66–1,227)	977 (196–2,683)	NA	NA
LVEF, %	65 ± 6	62 ± 6	63.5 ± 8.6	63.5 ± 8.6	62 ± 4	62 ± 6	NA	NA
	Shaltout 2017 (32)		Reddy 2017 (30)		Borlaug 2018 (28)		Francisco 2018 (29)	
	Treatment (*n* = 10)	Control (*n* = 9)	Treatment (*n* = 52)	Control (*n* = 52)	Treatment (*n* = 105)	Control (*n* = 105)	Treatment (*n* = 16)	Control (*n* = 16)
RCR design	Parallel-group		Parallel-group		Cross-over		Cross-over	
Intervention	A daily dose of BRJ (6.1 mmol of NO3^−^ in 70 ml) for 4 weeks		A single, acute dose of intravenous or inhaled sodium nitrite		Nebulized inhaled sodium nitrite, 46 mg three times daily for 1 week, and then 80 mg three times daily for 3 weeks		A single, acute dose of BRJ (12.9 mmol NO3^−^ in 140 ml)	
Intervention drug	Nitrate		Nitrite		Nitrite		Nitrate	
Control	Placebo		Placebo		Placebo		Placebo	
Treatment duration	Short-term		Acute		Short-term		Acute	
Exercise pattern	Constant 45-W exercise or maximal-effort exercise		Constant 20-W exercise		Upright cycle ergometry		NA	
White race, %	60	67	NA	NA	89	87	12.5	12.5
Age, yrs	68 ± 6.2	70.6 ± 7.6	62 ± 10	68 ± 12	68 ± 9	68 ± 12	65 ± 5.5	65 ± 5.5
Male, %	20	11	34.1	26.9	35.6	35	65	65
BMI, kg/m^2^	33.5 ± 5.8	31.5 ± 5.4	34.1 ± 7.9	26.9 ± 4.1	35.6 ± 6.4	35.0 ± 7.0	34.4 ± 3.5	34.4 ± 3.5
NYHA Class, %								
II	50	50	NA	NA	47	38	NA	NA
III	89	11	NA	NA	51	62	NA	NA
HTN, %	100	100	98	100	81	81	100	100
DM, %	50	20	31	23	38	33	68.8	68.8
CHD, %	NA	NA	35	27	68	71	18.8	18.8
Drug therapy, %								
Diuretic	70	56	43	13	88	94	62.5	62.5
ACEI/ARB	70	55	59	41	53	54	62.5	62.5
β-Blocker	30	22	58	50	60	67	62.5	62.5
Spironolactone	NA	NA	NA	NA	30	33	6.3	6.3
CCB	50	22	23	32	34	27	43.8	43.8
Statin	NA	NA	NA	NA	66	63	56.3	56.3
NT-proBNP, pg/ml	NA	NA	422 (122–1,022)	422 (122–1,022)	471 ± 624	528 ± 669	148	148
LVEF, %	NA	NA	63 ± 8	62 ± 9	51.4 ± 5	60.6 ± 6.7	62.4 ± 7.4	62.4 ± 7.4

RCT, randomized controlled trials; BMI, Body Mass Index; NYHA, New York Heart association; HTN, hypertension; DM, diabetes mellitus; CHD, coronary heart disease; ACEI, angiotensin-converting enzyme inhibitors; ARB, aldosterone receptor blockers; CCB, calcium channel receptor blockers; NT-proBNP, N terminal pro brain natriuretic peptide; LVEF, left ventricle ejection fraction; BRJ, beetroot juice; and NA, not available.

### Quality assessment

All studies included were prospective randomized controlled trials with relatively high quality. Through the evaluation of quality of evidence using the GRADE system (37), only two studies remained high quality. The other six studies all had different degrees of degradation and two were downgraded to a quality of very low. The results of quality assessment of the included studies were shown in Table 2.

**Table 2:** Grading of evidence quality of included studies according to GRADE.

References	Published year	Risk of bias	Inconsistency	Indirectness	Imprecision	Publication bias	Total	Quality of evidence
Borlaug (25)	2015	0	0	0	−1	−1	−2	Low
Zamani (21)	2015	0	0	0	0	0	0	High
Borlaug (26)	2016	0	−1	0	−1	−1	−3	Very low
Eggebeen (31)	2016	0	0	0	0	−1	−1	Moderate
Shaltout (32)	2017	0	−1	0	0	−1	−2	Low
Reddy (30)	2017	−1	0	0	0	−1	−2	Low
Borlaug (28)	2018	0	0	0	0	0	0	High
Francisco (29)	2018	−1	0	0	−1	−1	−3	Very low

### Primary outcomes

**Figure 2:**
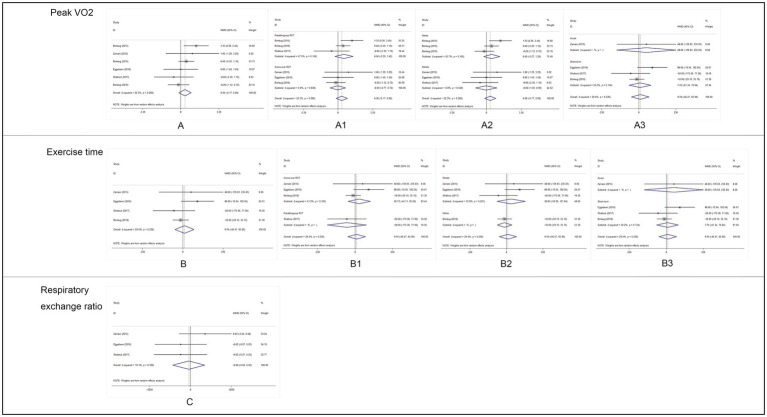
Analysis results of primary outcomes. **(A)** Forest plot of peak VO_2_, **(A**_**1**_**)**: subgroup analysis by RCT design, **(A**_**2**_**)**: subgroup analysis by intervention drug, and **(A**_**3**_**)**: subgroup analysis by intervention duration. **(B)** Forest plot of exercise time, **(B**_**1**_**)**: subgroup analysis by RCT design, **(B**_**2**_**)**: subgroup analysis by intervention drug, **(B**_**3**_**)**: subgroup analysis by intervention duration. **(C)**: Forest plot of respiratory exchange ratio (RER) during exercise.

**Figure 3:**
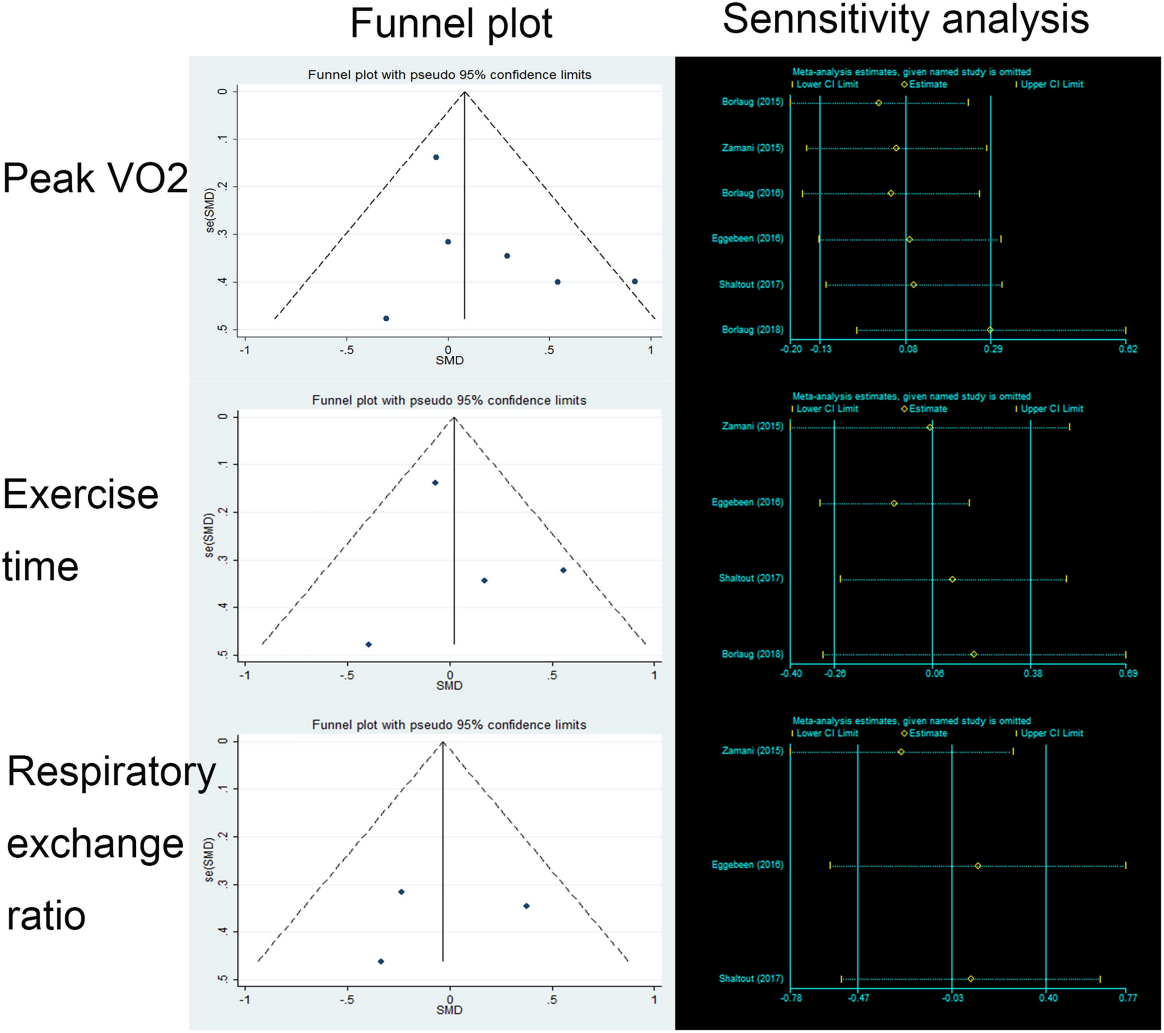
Funnel plot and sensitivity analysis of primary outcomes.

We found no benefit of inorganic nitrate/nitrite on exercise capacity in patients with HFpEF (Table 3). Inorganic nitrate/nitrite compared to placebo, did not significantly increased peak VO_2_ (MD = 0.361, 95% CI = −0.17 to 0.89, *p* = 0.183; Figure 2A), exercise time (MD = 9.74, 95% CI = −46.47 to 65.95, *p* = 0.734; Figure 2B), or either respiratory exchange ratio during exercise (MD = −0.003, 95% CI = −0.036 to 0.029, *p* = 0.834; Figure 2C). For peak VO_2_ and exercise time, subgroup analyses were conducted according to RCT design (parallel or cross-over), intervention subtype (nitrate or nitrite), and treatment duration (acute or short-term, also interpreted as “single administration” or “repeated administration”). As a result, no significant results were obtained for each subgroup (Figure 2). Neither nitrate (MD = 0.02, 95% CI = −1.03 to 0.99, *p* = 0.97) nor nitrite (MD = 0.50, 95% CI = −0.27 to 1.02, *p* = 0.20) was effective in increasing peak VO_2_ compared to placebo. Similarly, for exercise time, there was no difference either between nitrate and placebo (MD = 30.83, 95% CI = −50.99 to 121.64, *p* = 0.51) or between nitrite and placebo (MD = −12.0, 95% CI = −59.1 to 35.1, *p* = 0.62). As can be seen from our subgroup analysis according to treatment duration, the improvement of peak VO_2_ or exercise time was also absent in the acute effect of inorganic nitrate/nitrite (MD = 25.32, 95% CI = −19.24 to 66.73, *p* = 0.52; MD = 48, 95% CI = −139.83 to 235.83, *p* = 0.64). There was no subgroup analysis for respiratory exchange ratio due to its limited number of included studies. No statistically significant between-study heterogeneity was detected for primary outcomes. Further sensitivity analysis of each outcome showed that the exclusion of each study did not alter the significance of the corresponding pooled MD, suggesting that the results were robust. For publication bias, the shape of the funnel plot of each outcome was visually symmetric, then statistical assessment by Egger test suggested no significant publication bias in all pooled studies (*p* = 0.245; 0.681; 0.825; Figure 3).

**Table 3:** Synthesized SMD of outcomes.

Outcomes		Included studies (*n*)	Synthesized MD	*p* value	*I^2^* test (%)	Egger test
Primary outcome						
Peak VO_2_ during exercise, ml/min/kg		6	0.36 (−0.17, 0.89)	0.183	22.3	0.245
Exercise time, second		4	9.74 (−46.47, 65.95)	0.734	29.4	0.681
Respiratory exchange ratio during exercise		3	0 (−0.04, 0.03)	0.834	10.1	0.825
Secondary outcome						
Cardiac output, L/min	Rest	4	−0.09 (−0.31, 0.12)	0.402	15.4	0.044
	Exercise	4	0.42 (−0.37, 1.20)	0.298	81.2	0.017
Heart rate, beats/min	Rest	4	−0.39 (−3.90, 3.12)	0.83	78.5	0.017
	Exercise	6	0 (−0.74, 0.74)	0.993	0	0.726
Systolic BP, mmHg	Rest	4	−7.91 (−11.25, −4.56)	<0.001	14.4	0.054
	Exercise	5	−4.45 (−0.94, 0.53)	0.08	64.3	0.015
Diastolic BP, mmHg	Rest	2	−2.96 (−3.73, −2.20)	<0.001	0	–
	Exercise	4	−3.93 (−4.99, −2.82)	<0.001	0	0.309
MAP, mmHg	Rest	4	−2.91 (−3.65, −2.17)	<0.001	83.3	
	Exercise	3	−4.11 (−7.11, −1.11)	0.007	67.1	0.442
SVR, DSC	Rest	3	−23.96 (−103.91, 55.99)	0.557	21.9	0.322
	Exercise	4	−74.43 (−129.85, −19.01)	0.112	93.3	0.21

SMD, standard mean difference; BP, blood pressure; MAP, mean arterial pressure; SVR, systematic vascular resistance; and DSC, dyne second cm^−5^.

### Secondary outcomes

**Figure 4:**
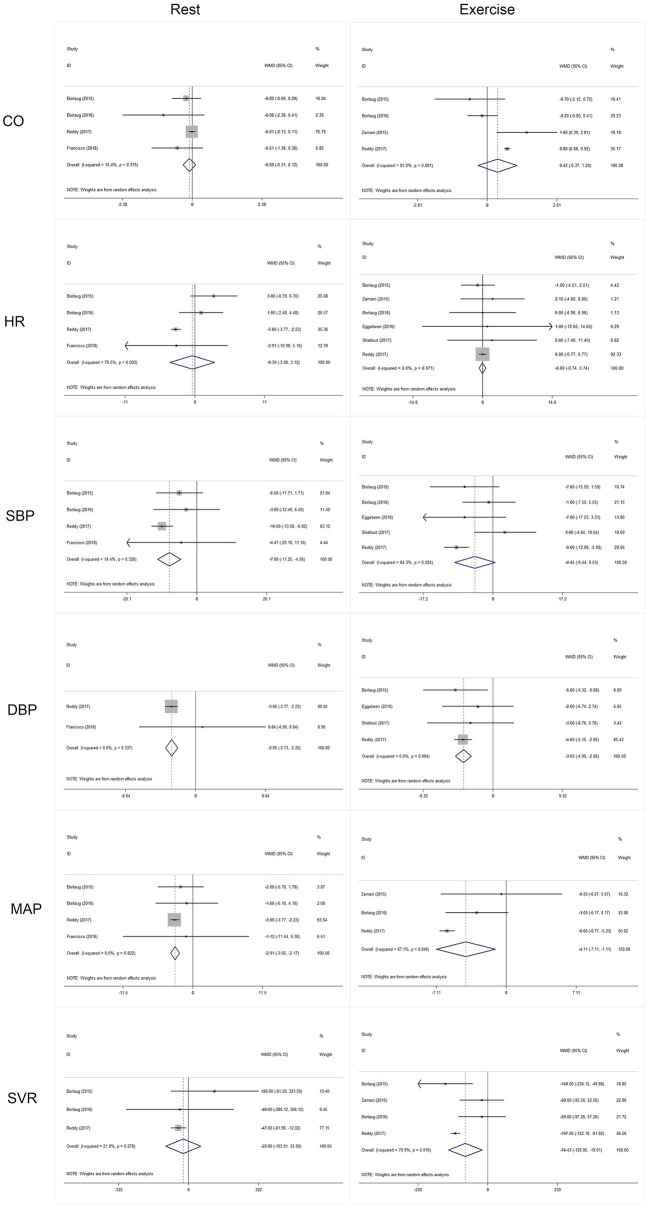
Statistical syntheses of secondary outcomes.

**Figure 5:**
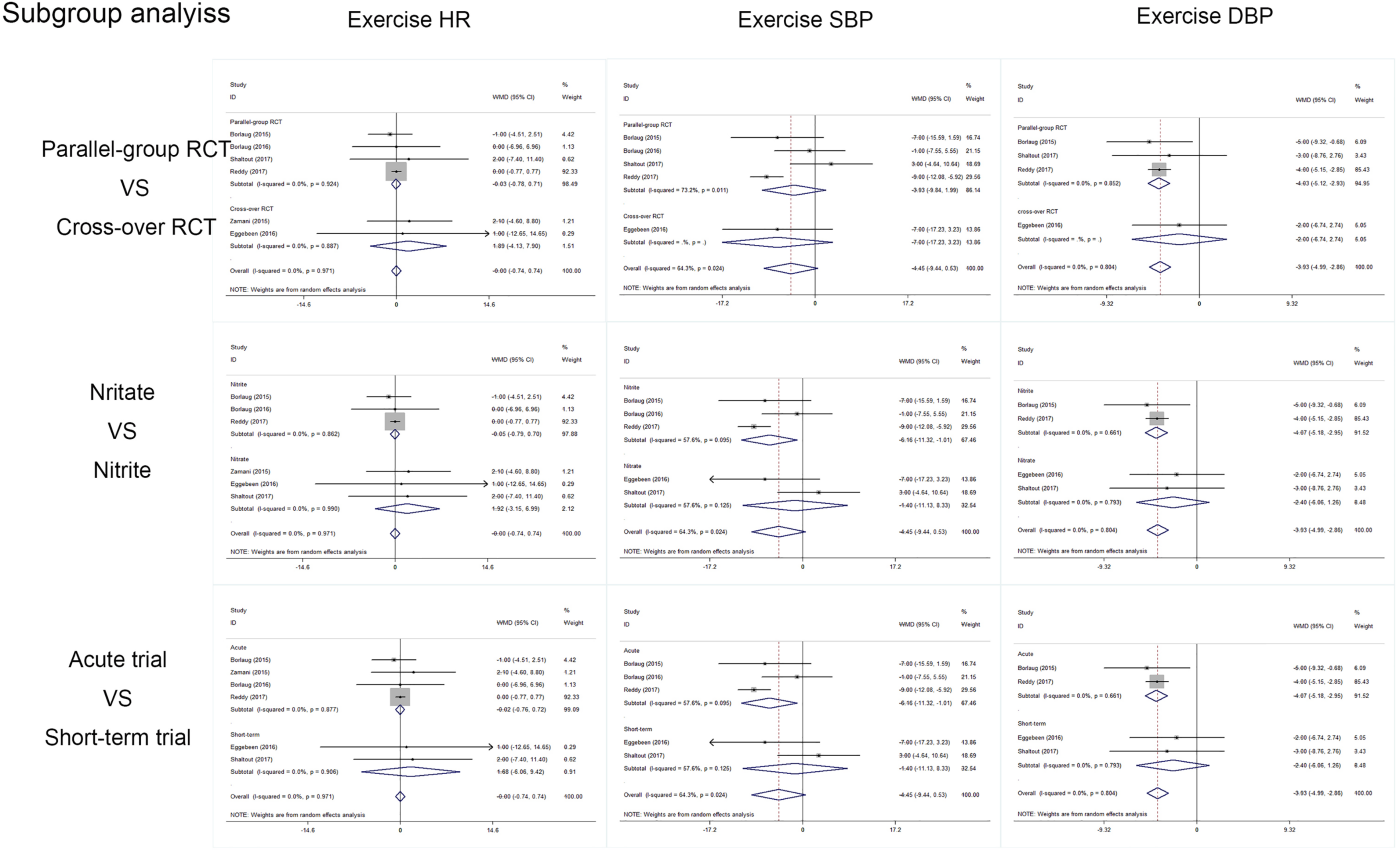
Subgroup analyses of secondary outcomes.

Outcomes of cardiac and artery hemodynamics diameters were pooled for analysis (Table 3). In general, we yield an equivocal result. Our analysis showed that compared to placebo, inorganic nitrate/nitrite can lower rest SBP (MD = −7.91, 95% CI = −11.25 to −4.56, *p* < 0.001), rest/exercise DBP (MD = −2.96, 95% CI = −3.73 to −2.20, *p* < 0.001; MD = −3.93, 95% CI = −4.99 to −2.82, *p* < 0.001), rest/exercise MAP (MD = −2.91, 95% CI = −3.65 to −2.17, *p* < 0.001; MD = −4.11, 95% CI = −7.11 to −1.11, *p* = 0.007), and exercise SVR (MD = −74.43, 95% CI = −129.85 to −19.01, *p* = 0.008) in patients with HFpEF. However, no significant difference was found for cardiac output and HR, either in rest or during exercise. Subgroup analyses by study design, intervention drug, and treatment duration were performed for outcomes of HR, SBP, and DBP during exercise. Consequently, there was still no significant difference in exercise HR in acute treatment (MD = −0.02, 95% CI = −0.761 to 0.723, *p* = 0.96) or short-term treatment (MD = 1.68, 95% CI = −6.06 to 9.42, *p* = 0.67), or in nitrate (MD = 1.92, 95% CI = −3.15 to 6.92, *p* = 0.46), or nitrite (MD = −0.045, 95% CI = −0.79 to 0.70, *p* = 0.91). All the general statistical syntheses of secondary outcomes were shown in Figure 4. However, the subgroup analyses for exercise SBP and DBP showed that it was nitrite (MD = −6.17, 95% CI = −11.32 to −1.01, *p* = 0.019; MD = −4.07, 95% CI = −5.18 to −2.95, *p* < 0.001) rather than nitrate (MD = −1.40, 95% CI = −11.13 to −8.33, *p* = 0.78; MD = −2.40, 95% CI = −6.06 to 1.25, *p* = 0.198) that was able to lower exercise blood pressure. Moreover, the antihypertensive effect induced by inorganic nitrate/nitrite appeared to be acute (or transient) rather than persistent, because the exercise SBP and DBP was significantly lowered only in the acute treatment (MD = −6.17, 95% CI = −11.32 to −1.01, *p* = 0.019; MD = −4.07, 95% CI = −5.18 to −2.95, *p* < 0.001) but no in the short-term treatment (MD = −1.40, 95% CI = −11.13 to −8.33, *p* = 0.78; MD = −2.40, 95% CI = −6.06 to 1.25, *p* = 0.198). All the general statistical syntheses of secondary outcomes were shown in Figure 5. Still, sensitivity analyses were conducted for each outcome, and no results were changed after any extraction of studies. In analyses for exercise/rest CO, exercise/rest SBP, and rest HR, publication bias on Egger test were found (*p* = 0.017, 0.044, 0.015, 0.054, and 0.017, respectively). However, further trim-and-fill test indicated that the estimates were not impacted by these publication bias (i.e., no trimming done because data unchanged).

## Discussion

The results of this meta-analysis show that compared with placebo, inorganic nitrate/nitrite therapy cannot improve peak VO_2_, respiratory exchange ratio, or exercise time, which means it has no benefits in improving the exercise capacity of HFpEF patients. However, analyses of hemodynamics indexes show that inorganic nitrate/nitrite can temporarily lower exercise blood in HFpEF.

When the mechanism for a disease is understood, the corresponding treatment comes into being. Although the mechanism of HFpEF has not yet been fully clarified, several major theories have been formed. Multiple lines of indirect trial evidence suggest that systematic microvascular inflammation plays an important role in the development of HFpEF (38–42). In HFpEF, microvascular inflammation caused by comorbidities reduces the availability of cyclic guanosine monophosphate (cGMP), thereby decreases nitric oxide (NO) activity and blocks actin phosphorylation, which ultimately damages adjacent cardiomyocytes (1). Accordingly, therapeutic trials aimed at the NO-cGMP pathway have been conducted as an investigation for treatment of HFpEF. Organic nitrates such as isosorbide mononitrate have been proved unable to lead a better quality of life or submaximal exercise capacity for patients with HFpEF (43). Furthermore, it may lead to hypotension due to excessive preload reduction and paradoxically cause endothelial dysfunction. In contrast to the organic nitrates that require aldehyde dehydrogenase and other enzymes for activation (44), there is no tolerance with nitrate–nitrite (23). Due to fewer side effects and enhanced pathway in the presence of hypoxia and acidosis, an increasing number of researchers are turning their attention to the field of inorganic nitrate/nitrite treatment for HFpEF. Although numerous trials have been conducted, the results have not been conclusive. This is why we conduct the current meta-analysis, and what we found may provide an answer for the current puzzle.

Exercise testing with ventilatory expired gas analysis has been acting as a valuable tool for assessing patients with heart failure (HF) (43–45). Peak exercise oxygen uptake (peak VO_2_), the standard for assessing cardiovascular fitness, plays an important role in prognosis and risk stratification among patients with chronic heart failure (CHF) (46–49). It is the gold-standard indicator of functional capacity and is depressed in patients with HFpEF (50–53). The elevated cardiac filling pressure in HFpEF result in reduction in peak VO_2_, promoting symptoms of dyspnea and limiting oxygen delivery, which ultimately impact exercise capacity. We therefore use peak VO_2_ as the primary indicator of exercise tolerance for patients in our study. Respiratory exchange ratio (RER), defined as the ratio of VCO_2_ to VO_2_, depends mostly on the skeletal muscle energy metabolism (54). In HF patients, overactivation of intramuscular ergoreceptors can induce excessive ventilatory response (i.e., hyperventilation), thereby yielding a reduced ventilatory efficiency with a higher RER even during submaximal exercise (55, 56). Reliable evidence suggested that high RER during exercise, particularly at anaerobic (AT) threshold workload, is associated with poor clinical outcome in HF patients (57). Consequently, the value of RER was used in our study as another important indicator to assess the exercise capacity. Finally, exercise time is the most intuitive indicator to evaluate patients’ exercise ability and the most important endpoint that intervention drugs need to target.

Our study set up three subgroup analyses based on RCT design, intervention drug, and intervention duration, respectively. We believe that such grouping can maximize the source of heterogeneity and minimize the inter-group heterogeneity, thus making the results more authentic. Considering that HFpEF is a chronic disease and has no self-healing tendency, many trials have applied cross-over design to better rule out the effects of confounding factors. Because the cross-over RCT possesses a higher level of evidence than the parallel-group RCT, it is necessary to separate the results from the two types of RCT. Then, although nitrate and nitrite both ultimately convert to NO through nitrate–nitrite–NO pathway (58–60), they are ingested in different ways. It is noteworthy that the ingested dietary NO_3_^−^ needs to be reduced to bioactive NO_2_^−^ by bacteria in the oral cavity before the NO_2_^−^ is taken up by the plasma from the digestive system and eventually converted to NO. In previous studies, inorganic nitrate was conformably ingested orally in the form of concentrated nitrate-rich beetroot juice (BRJ) (21, 29, 31, 32), whereas inorganic nitrite was supplemented using direct nitrite infusion (25) or inhalation (28). Indeed, previous studies focusing on inorganic nitrite (25, 26, 30) trended to drawn more significant results than that of nitrate (29, 31, 32), probably due to the more direct ingesting way of NO_2_^−^ than NO_3_^−^ and the first pass metabolism during the conversion from oral NO_3_^−^ to plasma NO_2_^−^ (31). Therefore, nitrate and nitrite studies were analyzed separately to investigate whether there is difference in therapeutic effect between the two subtypes. Another factor that induced huge difference between studies was the duration of administration, generally divided into single dose (to observe the instantaneous effect) and repeated doses (to observe the continuous effect) in previous studies. In the study by Eggebeen et al. (31), 1 week of daily dose with BRJ improved submaximal aerobic endurance, whereas no significant effect was found for this outcome with a single, acute BRJ dose. It could be explained by that the acute effects of inorganic nitrate/nitrite may be due to its instantaneous impact on cardiac hemodynamics, yet the long-term effects should depend on its chronic improvement on microvascular function. This is why a subgroup analysis was performed according to whether the administration was single, acute dose or repeated, chronic dose.

To our knowledge, this is the first meta-analysis assessing the inorganic nitrate/nitrite supplementary therapy for HFpEF. Despite the wealth of data showing favorable effects of therapies targeting the inorganic nitrate/nitrite pathway in HFpEF, our study did not find a positive effect of this treatment in exercise capacity. What we found is consistent with a recently published multicenter trial by Borlaug et al. (28), which demonstrated that inhaled sodium nitrite did not improved the clinical status of patients with chronic HFpEF. Our results are also in agreement with several previous studies showing that a dietary nitrate intake in the form of BRJ did not improve exercise intolerance or hemodynamics indicators such as mean arterial pressure, heart rate, or cardiac output in HFpEF patients, although the concentration and duration of BRJ intervention in these studies varied (29, 31, 32). The reasons for the discrepancies between the rationale that nitric oxide possesses the ability of improving microcirculation and cardiac function in patients with HFpEF and the absence of clinical benefit are not clear. Reasonable explanations might be that the half-life of plasma nitrite is too short to maintain a sustained high level of plasma cGMP, whose deficiency has been repeatedly shown to play a key role in the pathogenesis of HFpEF (1, 41). In addition, as each intervention ultimately works through the conversion of the NO_3_^−^–NO_2_^−^–NO pathway to the final effector—nitric oxide, whether the interventions in the included studies have caused a sufficient increase in circulating nitric oxide is unknown. Furthermore, the duration of the trials studied were relatively brief (up to 4 weeks), which might not have allowed adequate exposure to observe a favorable effect on cardiovascular structure and function. It is worth mentioning that multiple trials with longer duration of administration targeting the inorganic nitrate/nitrite pathway in HFpEF are currently underway (NCT03015402, NCT02980068, NCT02918552, NCT02840799, NCT03289481, and NCT02713126). Results of these trials are expected to provide stronger evidence, which may alter the current finding.

Multiple earlier studies have suggested that inorganic nitrate or nitrite has the trend of lowering arterial blood pressure, especially during exercise (25, 26, 30–32). Our findings confirmed this notion and further demonstrated that the reduction in blood pressure induced by inorganic nitrate/nitrite in HFpEF was instantaneous rather than continuous. It is widely noted that systemic hypertension is highly prevalent in HFpEF. However, whether arterial stiffening is more specific to HFpEF or just common to all hypertensive patients is unknown. For this reason, two earlier studies compared HFpEF subjects with carefully matched hypertensive control subjects in the measurement of arterial stiffness (61, 62). As a result, they both demonstrated that the elevations of arterial pressure and blood flow were more apparent during exercise when HFpEF subjects were compared to hypertensive control subjects, despite lack of discernable difference in resting arterial afterload, revealing that arterial stiffening plays an important role in the pathophysiology of HFpEF, especially during exercise. However, a prior study exploring the blood-pressure-lowering effect of dietary nitrate showed that a 4-week dietary nitrate could provide a sustained BP lowering in patients with hypertension (63), which contradicts with our finding that a long-term BP lowering effect is absent with inorganic nitrate/nitrite supplementation. It must be pointed out that the BRJ the patients received in that study was equal to 6.4 mmol nitrate, a daily dose higher than that of any our included studies. In addition, the arterial hemodynamics characters of HFpEF are more complicated than that of hypertension alone. More long-term outcome studies are needed to be carried out to explore the effect of inorganic nitrate/nitrite on BP lowering in HFpEF patients.

Taken together, our results can only support the role of inorganic nitrate/nitrite as an adjunct in the treatment of HFpEF, which seems to declare another drug to be ineffective for HFpEF, again. However, this does not mean our study is meaningless. On the contrary, our research is of great value for it is the first meta-analysis to fully summarize the clinical effects of inorganic nitrate/nitrite on HFpEF. Before this, inorganic nitrate/nitrite administration has long been considered as a promising new therapy being tested in HFpEF. As noted above, although a considerable number of studies have tested inorganic nitrate/nitrite in HFpEF, the question of whether it improves exercise capacity of HFpEF patients remains unanswered. In addition, our study provides a new direction for inorganic nitrate/nitrite treatment on HFpEF, in which thinking about how to administer the drug may be more useful than thinking about whether the drug is effective. Because from our results effects of different administrations of inorganic nitrate/nitrite may be quite different. For example, studies demonstrating positive results all used intravenous or inhaled nitrite, yet those demonstrating negative results almost used oral nitrate (BRJ), which suggests that the rate of administration and the efficiency of absorption greatly influence the therapeutic effect of inorganic nitrate/nitrite on HFpEF. Therefore, although our results generally showed ineffectiveness of inorganic nitrate/nitrite on HFpEF, attempts of different administrations are encouraged, and the question of the usefulness of inorganic nitrate/nitrite treatment can only be answered after enough evidence-based trials with various administrations are conducted.

Our study has several limitations. First, since we only enrolled a total of eight studies, the number of studies for the combination of a single outcome was relatively limited, which limited the persuasiveness of the results to some extent. Second, as we tried to include all the studies assessing therapies targeting inorganic nitrate/nitrite pathway, there was no restriction on the type and duration of intervention, which increased heterogeneity to some extent. To minimize the heterogeneity, we correspondingly conduct subgroup analyses. Third, with only three trials (26, 31, 32) measuring plasma nitrate/nitrite concentrations before and after administration, our study failed to analyze the relationship between elevated plasma nitrate/nitrite levels and intervention outcomes. In other words, whether the negative results we obtained was caused by the fact that the intervention did not cause sufficient elevation of plasma nitrate/nitrite or that elevated plasma nitrate/nitrite did not improve the exercise capacity of patients with HFpEF is unknown from our study.

## Conclusion

There is insufficient evidence to support the use of inorganic nitrate/nitrite for improving the exercise performance of patients with HFpEF at this time. But inorganic nitrite may yield a transient blood pressure lowering effect, especially during exercise. More prospective trials testing long-term effect of inorganic nitrate/nitrite therapy are warranted.

## Data availability statement

The original contributions presented in the study are included in the article/supplementary material, further inquiries can be directed to the corresponding author.

## Author contributions

FL and JZ: conceived the idea, data curation, and writing-original draft preparation. FL, JZ and YT: writing-review and editing. YT: supervision. All authors contributed to the article and approved the submitted version.

## Conflict of interest

The authors declare that the research was conducted in the absence of any commercial or financial relationships that could be construed as a potential conflict of interest.

## Publisher’s note

All claims expressed in this article are solely those of the authors and do not necessarily represent those of their affiliated organizations, or those of the publisher, the editors and the reviewers. Any product that may be evaluated in this article, or claim that may be made by its manufacturer, is not guaranteed or endorsed by the publisher.
